# Early Arrival and Climatically-Linked Geographic Expansion of New World Monkeys from Tiny African Ancestors

**DOI:** 10.1093/sysbio/syy046

**Published:** 2018-06-20

**Authors:** Daniele Silvestro, Marcelo F Tejedor, Martha L Serrano-Serrano, Oriane Loiseau, Victor Rossier, Jonathan Rolland, Alexander Zizka, Sebastian Höhna, Alexandre Antonelli, Nicolas Salamin

**Affiliations:** 1 Department of Biological and Environmental Sciences, University of Gothenburg, Carl Skottsbergs gata 22B, Gothenburg 41319, Sweden; 2 Department of Computational Biology, University of Lausanne, 1015 Lausanne, Switzerland; 3 Gothenburg Global Biodiversity Center, Carl Skottsbergs gata 22B, Gothenburg 41319, Sweden; 4 Swiss Institute of Bioinformatics, Quartier Sorge, 1015 Lausanne, Switzerland; 5 Instituto Patagónico de Geología y Paleontología (CCT CONICET-CENPAT), Boulevard Almirante Brown 2915, 9120 Puerto Madryn, Chubut, Argentina; 6 Facultad de Ciencias Naturales, Sede Trelew, Universidad Nacional de la Patagonia ‘San Juan Bosco’, 9100 Trelew, Chubut, Argentina; 7 Department of Zoology, University of British Columbia, 2212 Main Mall, Vancouver, BC Canada; 8 Division of Evolutionary Biology, Ludwig-Maximilians-Universität München, Großhaderner Straße 2, 82152 Munich, Germany; 9 Gothenburg Botanical Garden, Carl Skottsbergs gata 22A, 413 19 Gothenburg, Sweden; 10 Department of Organismic and Evolutionary Biology, Harvard University, 26 Oxford St., Cambridge, MA 02138, USA; 11 These authors contributed equally to this work; 12 These authors are severs as a co-last authorship

**Keywords:** Trait evolution, primates, birth-death models, Bayesian methods, fossils

## Abstract

New World Monkeys (NWM) (platyrrhines) are one of the most diverse groups of primates, occupying today a wide range of ecosystems in the American tropics and exhibiting large variations in ecology, morphology, and behavior. Although the relationships among the almost 200 living species are relatively well understood, we lack robust estimates of the timing of origin, ancestral morphology, and geographic range evolution of the clade. Herein, we integrate paleontological and molecular evidence to assess the evolutionary dynamics of extinct and extant platyrrhines. We develop novel analytical frameworks to infer the evolution of body mass, changes in latitudinal ranges through time, and species diversification rates using a phylogenetic tree of living and fossil taxa. Our results show that platyrrhines originated 5–10 million years earlier than previously assumed, dating back to the Middle Eocene. The estimated ancestral platyrrhine was small—weighing 0.4 kg—and matched the size of their presumed African ancestors. As the three platyrrhine families diverged, we recover a rapid change in body mass range. During the Miocene Climatic Optimum, fossil diversity peaked and platyrrhines reached their widest latitudinal range, expanding as far South as Patagonia, favored by warm and humid climate and the lower elevation of the Andes. Finally, global cooling and aridification after the middle Miocene triggered a geographic contraction of NWM and increased their extinction rates. These results unveil the full evolutionary trajectory of an iconic and ecologically important radiation of monkeys and showcase the necessity of integrating fossil and molecular data for reliably estimating evolutionary rates and trends.

Platyrrhines, or New World Monkeys (NWM), are a diverse group of primates currently distributed in the Neotropical region from Mexico to Northern Argentina but excluding the Caribbean islands. They are all arboreal, but exhibit a wide spectrum of locomotor postures as well as body sizes (Fleagle 2013). Although the taxonomic classification of platyrrhines is still being debated, platyrrhines are usually divided into three families: Atelidae (including howler, wooly, spider, and wooly spider monkeys), Cebidae (including squirrel monkeys and capuchins, and marmosets and tamarins), and Pitheciidae (titis, sakis, and uakaries) (Schrago et al. 2014; alternative classifications are discussed in the Appendix).

Platyrrhines are thought to have originated in Africa, from which they dispersed into South America probably during the middle or late Eocene. The oldest fossil record, described as *Perupithecus,* was recently discovered in western Peru (Santa Rosa), and is estimated to be of late Eocene age (c. 41–34 Ma) (Bond et al. 2015). The evidence for an African origin is supported by the exceptional morphological similarity between *Perupithecus* and the North African late Eocene *Talahpithecus*, which share almost identical diagnostic characters, such as cusps, crests, and basins in the upper molars (Bond et al. 2015). These findings reinforce the hypothesis of a trans-Atlantic dispersal event during the Eocene, probably by rafting on budding forest islets (Tarling 1980,Oliveira et al. 2009).

The fossil record of NWM is relatively diverse as compared with many other Neotropical animals (33 extinct genera; Tejedor and Novo 2017) but scarce in proportion to other mammals occurring in the same localities. In addition to *Perupithecus,* ancient records of platyrrhines also include Oligocene specimens from Contamana, Peru (Marivaux et al. 2016), and Salla, Bolivia, ca. 26 Ma (Hoffstetter 1969, Rosenberger et al. 1991, Takai et al. 2000, Kay 2015). Patagonian and Chilean forms are known from the Miocene, ca. 20-15.8 Ma (Tejedor and Novo 2017). These records conform to what is recognized as a first stage in platyrrhine evolution, with primitive and, in some cases, odd morphologies and often unclear phylogenetic positions (Rosenberger et al. 2009). It was not until the Middle Miocene of Colombia, in the renowned fossiliferous area of La Venta, that the crown platyrrhines started to evolve into anatomically more modern forms, with morphologies in some cases indistinguishable from some living genera (Hartwig and Meldrum 2002, Tejedor and Novo 2017).

Despite the fragmentary nature of the NWM’s fossil record, available paleontological evidence holds the potential to reveal the evolutionary history of the clade. In particular, the geographic localities where platyrrhine fossils have been found indicate that past populations expanded into the Caribbean (Hispaniola, Cuba and Jamaica, where they no longer exist), and as far south as Patagonia, the southernmost area where non-human primates ever lived (Tejedor et al. 2006, Kay 2015). Other important insights about platyrrhine evolution can be obtained from the body mass of extinct taxa, which is strongly linked (and therefore predictable) with the molar area (Gingerich et al. 1982, Conroy 1987). This allows a confident estimate of the body mass of extinct primates, even when the fossil record is extremely incomplete. The fossil record of platyrrhines shows that extinct taxa account for the largest (> 20 kg) and some of the smallest (~ 0.4 kg) taxa in the clade (as compared with the current range ~ 0.1–12 kg), but the evolutionary dynamics and underlying causes shaping this wide range of body sizes remain unclear.

Although the fossil record provides essential information about ancestral phenotypes and their evolution (Villaaña and Rivadeneira 2018), its inherent incompleteness is a limiting factor for understanding macroevolutionary processes (Hopkins et al. 2018). As an important complement to the fossil record, phylogenetic comparative methods based on molecular data and trait measurements of extant taxa can shed further light into evolutionary processes (O’Meara 2012). Phylogenetic comparative methods have substantially expanded our understanding of the pace and mode of phenotypic evolution across clades, showing instances of for example, pulsed evolution, adaptive radiations, and rate heterogeneity among lineages (Harmon et al. 2003, Hansen et al. 2008, Eastman et al. 2011, Venditti et al. 2011, Ingram and Mahler 2013, Uyeda and Harmon 2014, Duchen et al. 2017, Landis and Schraiber 2017). However, there are serious limitations to estimating ancestral states based on phenotypes of extant taxa only (Finarelli and Flynn 2006, Slater et al. 2012, Fritz et al. 2013, Slater and Harmon 2013). In particular, for quantitative traits, ancestral states inferred from extant taxa cannot be estimated to be outside the observed range and tend be an average of the observed values, without the possibility to infer evolutionary trends (Felsenstein 1985, Finarelli and Flynn 2006, Revell 2008). Although directional evolution is thought to be rare (Hunt 2007), it is well documented in the fossil record of several mammalian clades, including a trend towards larger body size in equids (Shoemaker and Clauset 2014) and a strong increase in brain volume in the hominin lineage (Seymour et al. 2016). These issues should be particularly important in modeling the evolution of NWMs, where the spectrum of body sizes and the geographic ranges are larger in extinct taxa than among living species.

Herein, we compile all available paleontological evidence and combine it with molecular and trait data from living species to infer the evolutionary history of NWMs. Based on a large molecular data set and comprehensive taxonomic information, we infer phylogenetic trees of extinct and extant lineages using the fossilized birth–death (FBD) method (Heath et al. 2014). We then analyze the history of two key traits in the evolution of platyrrhines: body mass and the mean latitude of their geographic range. The abundance of teeth in the fossil record, as compared with other skeletal parts, allows us to infer body mass for all described extinct taxa. The location of the fossil sites also provides valuable information on the geographical evolution of the clade. We develop a Bayesian framework to infer the evolutionary history of quantitative traits using phylogenies that incorporate both extant and extinct lineages, which we validate through extensive simulations. The method allows us to jointly estimate the ancestral states at each node in the tree and the rate (describing how fast a trait evolves) and trend (the tendency of a trait to evolve in a certain direction, for example, toward larger or smaller values). Both rate and trend parameters can change across lineages in the tree, and our algorithm jointly estimates the number and placement of shifts in parameter values. Finally, we investigate how the speciation and extinction rates underlying NWMs diversification relate to their geographic and phenotypic changes. To this end, we expand the implementation of FBD process to infer speciation and extinction rates and their temporal variation while accounting for extinct and extant taxa.

## Methods

### Bayesian Analysis of Trait Evolution

**Figure 1.**
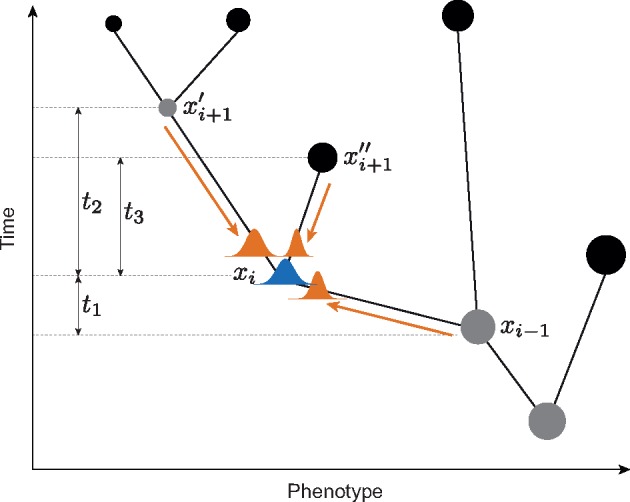
Posterior density of ancestral states. Ancestral states at the internal nodes of the tree are sampled directly from their posterior distribution, which combines three normal densities: two from the descendants and one from the parent node (all of which are based on the current trait states and parameters of the BM model), resulting in a normal posterior distribution (blue graph). The notation follows that of Eq. 2.

We implemented a Bayesian algorithm to estimate the evolutionary history of quantitative traits in a phylogenetic framework. The main parameters we aim to infer are 1) the rate and directionality of phenotypic evolution, 2) the heterogeneity of rates and directionality across the branches of the tree, and 3) the ancestral states at all internal nodes of the tree. The evolutionary models implemented here are based on Brownian motion (BM) in which the expected trait value $v_{i+t}$ at time $t$ follows the normal distribution:

(1) \begin{equation*} v_{i+t}\sim \mathcal{N} (v_i+\mu_0t, \sigma^2t), \end{equation*}

where $v_{i}$ is the ancestral trait value at time $i$, $\mu $ is the trend parameter describing the tendency of a trait to evolve in a direction, and $\sigma^{{2}}$ is the rate parameter describing the speed of phenotypic change. Note that most commonly neutral BM models are applied in the absence of fossil data by setting $\mu_0 = 0$. In our implementation, we relax the assumption of a constant BM model by allowing both the rate and the trend parameters to vary across clades in the phylogeny. We use a Bayesian algorithm to infer the number of rates and trend parameters from the data (see below and Supplementary Text available on Dryad at http://dx.doi.org/10.5061/dryad.sv43650). In addition to the BM model parameters, our approach jointly estimates the ancestral states of the quantitative trait for all internal nodes. The likelihood of a vector of ancestral states ***v***$=$ [$v_{1}$*, … v*$_{N-1}$] (where $N$ is the number of extinct and extant tips in the tree) is calculated as a product of normal densities based on Equation 1 and on the current values of ancestral states and BM parameters, recursively from the tips to the root (Fig. 1) (Felsenstein 1985).

In our model, all descendant species following a shift inherit the same rate or trend parameter, which is treated as independent of the parameters in the other branches of the tree. The number of parameter shifts (rates or trends) for a phylogeny of $n$ tips can therefore range between 0, that is, a homogeneous Brownian model of evolution, and the number of nodes in the phylogenetic tree excluding the root ($n-$2). To infer the number and placement of shifts, we used Birth–Death Markov chain Monte Carlo (MCMC) (BDMCMC) (Stephens 2000), an algorithm that has been previously used to estimate rate shifts in other stochastic processes in an evolutionary biology context (Silvestro et al. 2014). Unlike the reversible-jump MCMC (Green 1995), the BDMCMC-moves across models are not based on an acceptance probability, but on a stochastic birth–death process that adds or remove parameters from the model. The birth rate determines the probability of proposing a new shift in rates or trends and is fixed to one (Stephens 2000), whereas individual death rates are calculated for each class of parameters defined by a shift. Death rates determine the probability of removing a rate or trend shift. We use a Poisson distribution with shape parameter set to one as prior distribution on the number of rate and trend shifts. We compute the death rate of a shift by calculating the likelihood of the trait under a BM model with and without the shift. To compute the likelihood without a shift we set the rate (or trend) of the clade identified by the shift to the background rate (or trend), that is, the current parameter value at its parent node. The death rate of a parameter class is computed as the ratio between the likelihood without the shift and the likelihood with the shift (Stephens 2000, Silvestro et al. 2014). Thus, rate or trend shifts that improve the fit of the model have a very low extinction rate, and are unlikely to be removed during the BDMCMC. In contrast, rate shifts that do not improve the tree likelihood (or even decrease it) result in high extinction rates, and will be removed very quickly by the BDMCMC algorithm.

The algorithm starts with the simplest BM model (i.e., with homogeneous rate and trend parameters) and randomly selects a clade (with equal probability across all clades) for which a new rate or trend is sampled from their prior distribution. In this case, we use an exponential distribution for rates and a normal distribution centered in 0 and standard deviation set to 1 for trends. The introduction of a shift in the model represents a “birth” event. As soon as there is at least one shift, the death rates for each clade identified by a shift are calculated and the following event of the birth–death process will be determined by the relative magnitude of the rates. Additional details about the BDMCMC algorithm are described by (Stephens 2000, Silvestro et al. 2014).

For a given set of rate and trend parameters and a vector of ancestral states, the likelihood of a BM model can be calculated as a product of normal densities moving from the tips to the root. We sampled the rate and trend parameters and the ancestral state at the root of the tree using MCMC with acceptance probabilities defined by the posterior odds and the Hastings ratio (Metropolis et al. 1953, Hastings 1970). We used multiplier proposals for the rate parameters (while properly adjusting the Hastings ratio) and sliding window proposals for trends and the root state.

Sampling the ancestral states from their posterior distribution using the typical acceptance ratio of a Metropolis-Hastings MCMC can be difficult due to the large number of parameters (one for each internal node in the tree), which increase linearly with the number of tips. Thus, we implemented a Gibbs sampler, in which the ancestral states are sampled directly from their posterior density. This is possible because the posterior probability distribution of an ancestral state under a BM model is itself normally distributed (Felsenstein 2005). Indeed, because the expected trait value of a BM model after a time $t$ is normally distributed (Equation 1), the posterior density of an ancestral state $x_{i}$ derives from the combination of three normal distributions. To sample the ancestral states from the posterior we therefore draw random values from the conjugate distribution:

(2) \begin{eqnarray*} &&\underbrace{x_{i}}_{\rm posterior} \sim\underbrace{\mathcal{N}(x_{i-1}+\mu_{0}t_{1},\sigma^{2}t_{1})}_{\rm ancestor} \,\times\, \underbrace{\mathcal{N}(x_{i+1}'-\mu_{0}t_{2}, \sigma^{2}t_{2})}_{\rm decendant\ 1}\nonumber\\ &&\quad \times\, \underbrace{\mathcal{N}(x{''}_{i+1}-\mu_0t_3,\sigma^2t_3)}_{\rm decendant\ 2} \end{eqnarray*}

where $x_{i}$ is the trait value at a node $i$, $x_{i-1}$ is the trait value at $i$’s parent node, $x'_{i-1}$ and $ x''_{i+1}$ are the trait values at the two descendent nodes, $t_{{1-3}}$ are the branch lengths separating the nodes (Fig. 1), and $\mu_0$ and $\sigma^{{2}}$ indicate the trend and rate parameters, respectively. In our implementation, a Gibbs move implies updating all ancestral states iteratively, sampling from Equation 2.

We used an exponential prior on the rate of trait evolution $\sigma ^{{2}}$, and a normal distribution (with mean $=$ 0 and standard deviation $=$ 10) on the trend parameters. We thoroughly tested our implementation through extensive simulations (see Supplementary Information available on Dryad) assessing 1) the robustness of model selection using BDMCMC, 2) the accuracy of parameter estimation, 3) the effect of incomplete taxon sampling, and 4) the performance of our algorithm compared to alternative implementations.

### Phylogenetic Analysis of Extinct and Extant Platyrrhines

We used the molecular data set from Springer et al. (2012) from which we kept only the 87 species of platyrrhines (i.e., 44% of the know platyrrhine present diversity; Rylands and Mittermeier 2009) and discarded all markers for which the data coverage was below 30%. The reduced alignment included 54 nuclear genes and one mitochondrial gene for a total length of 36,065 bp, with average coverage per gene of 60% of the taxa (Supplementary Tables S2 and S3 available on Dryad). We used 34 fossil taxa with ages ranging from the Late Eocene to the Pleistocene (Supplementary Tables S4 and S5 available on Dryad) to infer a phylogeny of living and extinct platyrrhines. We jointly estimated phylogenetic relationships and divergence times in BEAST v2.4.3 (Bouckaert et al. 2014) under the FBD model implemented in the Sample Ancestor package (Gavryushkina et al. 2014, Heath et al. 2014). We selected a log-normal relaxed clock and used the same gene partitions as in Springer et al. (2012) and selected GTR$+\Gamma $ substitution models for each partition after model-testing using j-Modeltest (Posada 2008) (Supplementary Tables S4 and S5 available on Dryad). Under the FBD model, fossil taxa can be treated as direct ancestors or extinct tips and their topological placement is integrated out using MCMC (Heath et al. 2014). We used taxonomic information following Rosenberger et al. (2009) and Tejedor and Novo (2017) to constrain the placement of extinct taxa in the phylogeny when possible e.g., to a family or subfamily; Supplementary Table S4 available on Dryad; Appendix). To sample phylogenetic trees of extinct and extant species from their posterior distribution, we ran two MCMC analyses for 100 million generations, sampling every 10,000 generations. We examined both runs in Tracer v1.5 to check for convergence and combined the two runs after removing a burn-in of 25 million generations. The resulting effective sample sizes were well above 100 for all parameters (median ESS $=$ 3066).

### Trait Analyses

We compiled fossil data and body mass estimates for most extant and extinct taxa from (Fleagle 2013). We additionally obtained data for *Perupithecus* from (Bond et al. 2015), for *Canaanimico* from (Marivaux et al. 2016), for *Talahpithecus* from (Jaeger et al. 2010), and for *Panamacebus* from (Bloch et al. 2016).

We computed mean latitudes of extant species from their latitudinal ranges as defined in the IUCN database (http://wwwiucnredlist.org) when available, or in the Pantheria database (Jones et al. 2009) otherwise. Because most fossil taxa are known from single localities, we used the latitude or their sampling locality as representative of their mean latitudinal range. We treated latitude as a quantitative trait to infer temporal changes in the ancestral distribution in platyrrhine evolution (e.g., Duchêne and Cardillo 2015).

We ran trait evolution analyses on 100 trees randomly selected from the BEAST posterior sample in order to incorporate topological and temporal uncertainties in our estimates. On each tree, we ran 5 million BDMCMC iterations sampling every 5000. Because the trees differed in branching times and in topology, we summarized the ancestral states for each family and subfamily (Supplementary Tables S11 and S12 available on Dryad), which form highly supported monophyletic groups (Supplementary Fig. S4 available on Dryad). We also calculated the range of trait values occupied through time as the minimum and maximum boundaries of the range of estimated ancestral states averaged over 100 analyses within 1 million-year time bins, following the procedure of (Serrano-Serrano et al. 2015). The number and placement of rate and trend shifts estimated through BDMCMC varied across trees. Thus, we summarized the parameters by families and subfamilies, by averaging the estimated rates and trends across the lineages within (sub)families. For comparison, we repeated the analysis of body mass and mean latitude evolution after dropping all extinct taxa from the platyrrhine phylogeny (Supplementary Figs. S8 and S9 available on Dryad).

### Diversification Rate Analysis

The original implementation of the FBD process (Heath et al. 2014) allows a joint estimation of speciation, extinction, and fossilization rates from a phylogenetic tree of extinct and extant taxa, assuming time-homogenous birth–death and preservation processes. We expanded the FBD model to incorporate temporal variation in speciation and extinction rates, thus allowing us to infer whether the diversification dynamics underlying platyrrhine evolution have changed or remained constant through time. We developed an Episodic-FBD (EFBD) model, where rates of speciation and extinction are constant within small time intervals, but can vary across intervals (see detailed description in the Supplementary Information available on Dryad). The model explicitly corrects for incomplete sampling of the extant taxa based on a user-defined sampling fraction (Supplementary Information available on Dryad). We implemented the EFBD model in RevBayes (Hohna et al. 2016) and ran an MCMC simulation for 50,000 iterations (each iteration consisted of 383 moves) to approximate posterior distribution of the parameters, sampling every 10 iterations. We repeated the analysis on a distribution of 100 trees and summarized the results by computing the posterior mean rates and 95% credible intervals (CI) through time for all parameters (Fig. 5). The complete list of priors and proposals used in these analyses are provided in Supplementary Tables S14 and S15 available on Dryad. For comparison, we repeated the diversification rate analysis after dropping all extinct taxa from the platyrrhine phylogeny (Supplementary Fig. S11 available on Dryad).

### Data and Software Availability

All the data used in this study (including the nucleotide alignment, BEAST input file, and trait data) are available here: https://github.com/dsilvestro/fossilBM. The repository also includes all the R and Python scripts developed to simulate data and analyze them. The EFBD model is available in RevBayes v1.0.7 from https://github.com/revbayes/revbayes and https://www.revbayes.com.

## Results

### Trait Evolution: Methodological Validation

**Figure 2.**
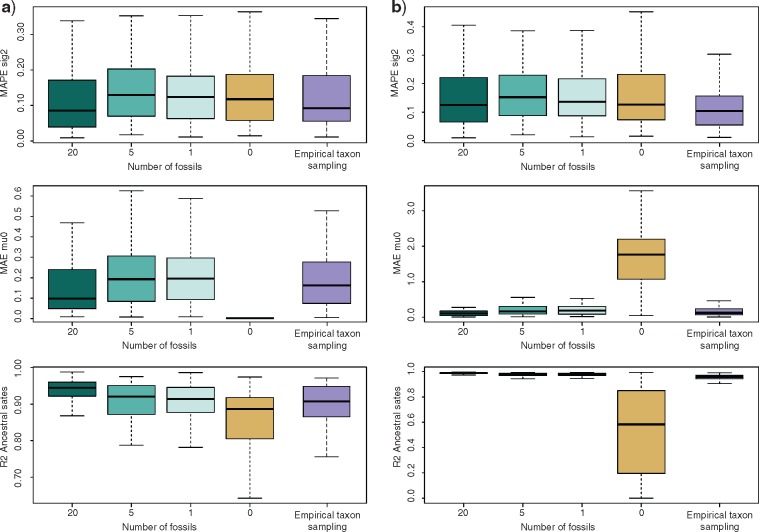
Accuracy of parameter estimation summarized across 100 simulations under Scenarios 1 and 2 and different levels of sampling***.* –** The simulations assumed a constant rate Brownian evolution and either no trend ($\mu_0 = 0$, A) or a positive or negative evolutionary trend ($\mu_0\ne$ 0; B). We tested decreasing number of fossils (20, 5, 1, and 0) and incomplete taxon sampling at the present. The latter setting (purple plots) was based on trees that reflected the size and sampling of the platyrrhine phylogeny analyzed here (200 extant taxa, 44% of which are sampled and 20 fossils). When the number of fossils was set to 0, only extant taxa were included in the analysis and the trend parameter ($\mu_0)$ was not estimated but set to 0.

Extensive simulations show that our novel Bayesian method for inferring trait evolution along a phylogeny with extinct and extant taxa provides accurate estimates of the rate and trends parameters and their variation across simulated clades (Supplementary Fig. S2 available on Dryad). Our algorithm correctly identified the number of shifts in rate or trend (if any) with an average frequency of 91.5% (ranging from 79 to 98% across different simulation settings; Supplementary Table S8 available on Dryad). The mean absolute percentage error (MAPE, see Methods section) ranged from 0.11 to 0.22 for the rate parameter and the mean absolute error (MAE) between 0.11 and 0.23 for the trend parameter, in data sets with 20 fossils included, that is, fewer than those included in empirical analyses of platyrrhines (Fig. 2 and Supplementary Fig. S1 available on Dryad). The estimated ancestral states were accurately estimated with average coefficients of determination ($R^{{2}})$ ranging from 0.92 to 0.99 across simulations (Fig. 2 and Supplementary Fig. S1c available on Dryad). Decreasing the number of fossils included in the data had small effects on the accuracy of the estimated rate and trend parameters and on the ancestral states (Fig. 2). In the absence of fossil data, the method could still estimate accurately the rate parameter (while the trend parameter is unidentifiable), but the accuracy of the ancestral states decreased to $R^{{2}} = 0.82$ for data sets simulated without trends, and to $R^{{2}}= 0.53$ for traits evolving under a non-zero trend (Fig. 2). Simulations which reflected the size and taxon sampling of the platyrrhine phylogeny analyzed here, resulted in accurate estimates of the rate parameters (MAPE $=$ 0.12), trend parameters (MAE $=$ 0.19), and ancestral states ($R^{2}$ > 0.90; Fig. 2). These results indicate that the method is robust to random incomplete sampling.

The performance of our algorithm in terms of efficiency of sampling the parameters from their posterior distribution and time to evaluate the likelihood significantly outperformed traditional algorithms, reducing computation times by one order of magnitude (Supplementary Fig. S3 available on Dryad). Notably, whereas computation time of common alternative implementations increases exponentially with tree size (e.g., Harmon et al. 2008, Revell 2012, but see e.g., Ho and Ane 2014), it increases linearly with our method, thus allowing for efficient analysis of very large data sets (thousands of tips; Supplementary Fig. S3 available on Dryad). The speed-up is due to 1) the way the likelihood is computed (a product of normal densities instead of operations based on a variance-covariance matrix) and 2) with the use of a Gibbs sampler to draw ancestral states directly from their posterior distribution (see Supplementary Text*:* Performance tests available on Dryad).

### NWM Phylogeny

Our phylogenetic analysis of the platyrrhine clade encompassed 87 extant species and 34 extinct taxa, spanning from the Late Eocene to the Holocene (Supplementary Tables S6 and S7 available on Dryad). The phylogenetic relationships between extant species were highly supported (Supplementary Figs. S4 and S5 available on Dryad) and reflected previous findings (Springer et al. 2012), while the placement of extinct taxa was sampled by the FBD within the limit of taxonomic constraints (see Methods section). The FBD analysis placed the origin of the clade in the Eocene and the divergence times of families and subfamilies between the late Oligocene and the mid-Miocene (Table 1).

**Table 1.** Estimated branching times summarized for the main clades within platyrrhines

Clade	Age (Ma)	95% Credible interval
Origin of the clade	43.53	37.64–50.77
Crown age of Platyrrhini	32.84	27.43–38.60
Crown age of Pitheciidae	25.11	21.01–28.88
Crown age of Cebidae	24.05	20.98–27.29
Crown age of Cebinae	22.33	20.93–25.00
Crown age of Pitheciinae	21.76	20.00–25.10
Crown age of Aotinae	20.91	20.00–23.69
Crown age of Homunculinae	19.49	17.00–23.76
Crown age of Atelidae	18.36	14.26–22.58
Crown age of Callitrichinae	17.60	14.38–20.82
Crown age of Alouattinae	15.09	12.50–18.29
Crown age of Atelinae	11.99	8.96–15.47

*Notes:* The origin of the clade corresponds to the stem age of platyrrhines, while crown ages indicate the ages of the most recent common ancestor of living representatives of each subclade, as estimated by the fossilized birth–death model.

### Body Mass

**Figure 3.**
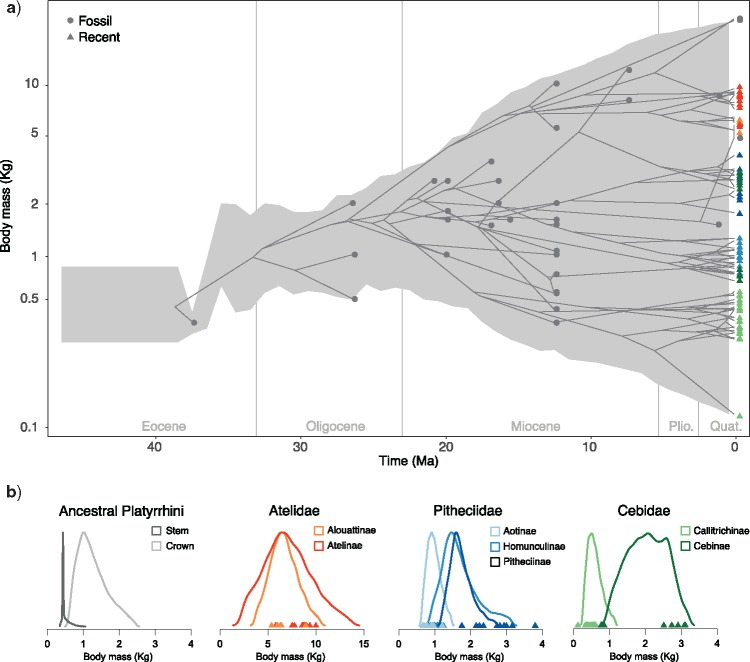
Body mass evolution in New World Monkeys. A) The gray shaded area shows the range of trait values (log-transformed body mass) through time (95% credible interval) inferred across a sample of 100 phylogenies with extinct and extant taxa (one of which was chosen randomly as an example). The grey circles indicate the body mass and age of the fossil taxa included in the analysis, and the blue triangles show the log body mass of extant species. B) The density plots show the estimated ancestral body mass (posterior distributions truncated to their 95% credible intervals) across subfamilies, for the most recent common ancestor of extant platyrrhines and at the root of the tree.

Platyrrhine body mass was estimated to have evolved under a BM model with variable rates and little or no evidence for positive or negative trends. The estimated number of rate shifts was 2 (95% CI: 1–4 shifts), whereas the trend parameter was found to be homogeneous across branches (Supplementary Table S10 available on Dryad). We found relatively high rates of body mass evolution at the family level, compared with substantially lower rates within subfamilies, except for subfamily Cebinae, for which a high rate was inferred (Supplementary Fig. S6 available on Dryad). The trend parameters were overall very close to 0, indicating non-directional evolution of body mass. The ancestral body mass inferred at the root of the tree ranged between 260 and 890 g (Fig. 3; Supplementary Table S11 available on Dryad). This estimate strongly differs from the estimate obtained from a phylogeny of extant NWMs, after discarding the fossil record (Supplementary Fig. S8 available on Dryad), where the estimated ancestral body mass at the root ranged between 949 and 2710 g (Supplementary Table S11 available on Dryad).

### Range Occupancy

**Figure 4.**
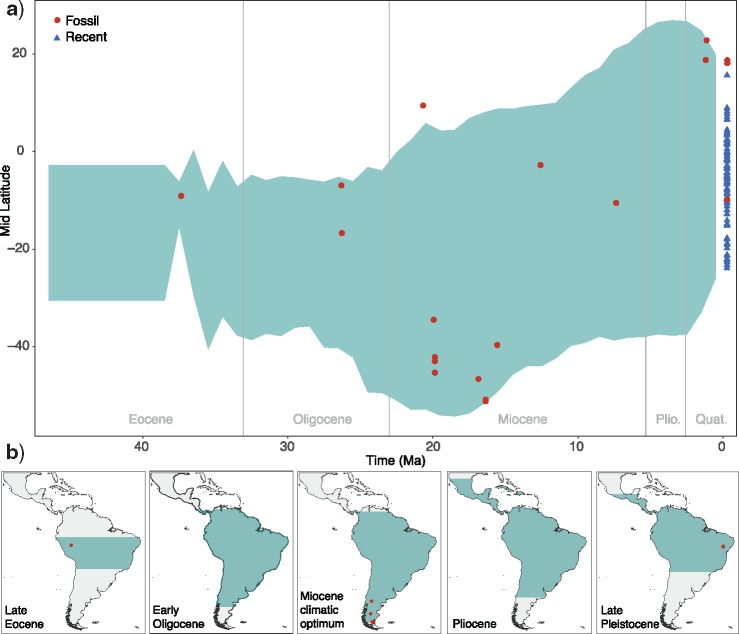
Changes in latitudinal ranges. A) The green shaded area shows the span of mean latitudinal ranges through time (95% credible interval) inferred across a sample of 100 phylogenies with extinct and extant taxa. The red circles indicate the (present day) latitude of the localities of the fossil taxa included in the analysis, and the blue triangles show the mid latitude of the geographic range of extant species. B) The histograms show the estimated ancestral latitudinal ranges projected onto the map of modern Central and South America.

Latitudinal ranges in platyrrhines have changed at variable rates across lineages. We estimated 3 rate shifts (95% CI: 1–4 shifts) among the 100 FBD phylogenies analyzed (Supplementary Table S10 available on Dryad). The rates were highest in the subfamilies Alouattinae and Cebinae (Supplementary Fig. S7 available on Dryad). The trend parameter was inferred to be constant across clades and positive, although a trend of 0 (i.e., no trend) was included in the 95% CI (Supplementary Table S12 and Fig. S7 available on Dryad). The inferred ancestral latitudes through time show an expansion to the South which culminates in the Early Miocene. The slightly positive trend likely captures the general trend of ancestral latitudes towards the current-day tropical zone following the disappearance of platyrrhines from the south of South America around the Middle Miocene and expansion into Central America during the Miocene and Pliocene (Fig. 4 and Supplementary Fig. S7 available on Dryad).

### Diversification Rates

**Figure 5.**
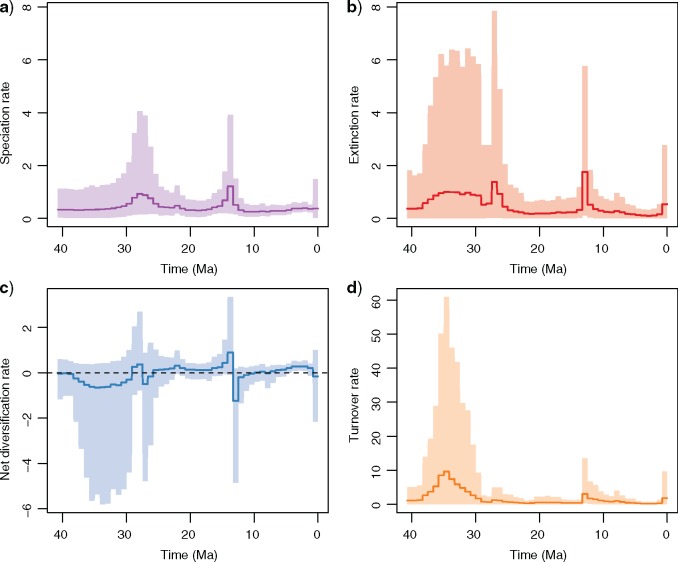
Diversification rates through time. Results of the EFBD analysis. Speciation and extinction rates (A and B) indicate the estimated number of speciation or extinction events per lineage per Myr. Net diversification rate equals speciation minus extinction rates and the turnover rate equals extinction divided by speciation rates (C and D). Dark lines indicate mean posterior estimates and shaded areas show the 95% credible intervals. Large credible intervals in the early stages of platyrrhine evolution indicate significant uncertainty around their diversification dynamics in that phase. During the MCO (18–15 Ma), we infer a peak in speciation rate followed by a peak in extinction rate, which result in negative net diversification. This event is coincident with global cooling and local environmental changes in the south of South America and with a geographic contraction of platyrrhine geographic range.

The analysis of phylogenies of extinct and extant taxa using our new EFBD model show that diversification rates have varied throughout NWM’s evolution (Fig. 5). Rate estimates are blurred by very large credible intervals in the Eocene and Oligocene, where the phylogenetic and fossil data available are limited, potentially also reflecting low platyrrhine diversity at the time. NWM underwent positive net diversification during the first half of the Miocene with a peak around 14 Ma, after which net diversification rates rapidly dropped to negative values (Fig. 5C). This pattern was driven by changes in both speciation and extinction rates. We inferred a four-fold increase in speciation rate (compared to the rate in the early Miocene) culminating ~ 14 Ma and decreasing almost five fold quickly around 13 Ma (Fig. 5A). Additionally, we detected a sudden nine fold increase in extinction rates around 13 Ma (Fig. 5B). A second, smaller, drop in diversification due to increased extinction is inferred to take place during the late Pleistocene (the last 1 Myr).

## Discussion

### Origin and Early Evolution

Our phylogenetic analysis of the platyrrhine clade inferred generally older estimates of divergence times compared to previous studies (Springer et al. 2012, Perez et al. 2013, Kay 2015, Bloch et al. 2016). For instance, the crown age of all extant platyrrhines is pushed back from the Early Miocene of previous estimates to the Early Oligocene. This is likely the result of using a larger and more complete fossil data set and a more realistic approach to calibrate the tree (Heath et al. 2014, Saladin et al. 2017). The estimated time of origin (stem age) of the platyrrhine lineage is 43 Ma (95% CI: 37.64–50.77 Ma). This indicates that the morphologically similar but now extinct North African anthropoids are comparable in age (Seiffert 2012) (Supplementary Table S10 available on Dryad). This finding suggests that platyrrhines (or pre-platyrrhines) evolved first in Africa, where they eventually went extinct, and dispersed sometime in the Eocene into South America where they diversified, in the absence of other primates on the continent (Bond et al. 2015). Previous estimations also suggested an Eocene age for the catarrhine-platyrrhine split (Perez et al. 2013, Schrago et al. 2014). Based on these lines of evidence, it is probable that the NWMs origin dates back to at least the Middle Eocene.

Ancestral state estimates indicate that NWMs derive from a remarkably small ancestor (around 400 g; Fig. 3), with an inferred body mass close to the lower boundary of the size range of extant platyrrhines (currently, only 11 species weigh less, see data sets in https://github.com/dsilvestro/fossilBM). In addition to *Perupithecus*, the oldest fossil included in this study, fossil records from the same locality include two broken upper molars and a lower molar of unidentified primate taxa (Bond et al. 2015). Their damaged condition did not allow an accurate description of these taxa, and the specimens were therefore, not included in our analyses. However, their approximate body size was likely around 70% of the size of *Perupithecus*. Thus, the taxon was possibly the size of a living tamarin, such as *Callimico* or *Saguinus*, weighing about 280 g. Despite the uncertainties around these estimates, this fragmentary fossil evidence indicates that the body mass of the ancestral NWM might have been even smaller than 400 g and closer to the size of small marmosets *Mico*, *Callithrix,* and *Cebuella*. The hypothesis of a small ancestral body mass at the origin of NWMs is also supported by the estimated size of Eocene anthropoids, especially parapithecoids from North Africa, which are mostly around 500 g (Jaeger et al. 2010, Fleagle 2013), with *Talahpithecus* weighing less than 400 g (Supplementary Table S13 available on Dryad). We propose that a small body size was probably a key factor enabling the survival of the individuals that reached South America from Africa, since their resource requirements on a floating islet would have been substantially smaller (e.g., relying on a diet of invertebrates and being better capable of protecting themselves against dehydration) than for a heavier and larger organism.

### Increase in Body Size

NWMs reached larger body sizes above 2 kg between the Late Oligocene and Early Miocene. Then, the upper bound of the body mass range in NWMs continuously increased, eventually reaching now extinct giant forms in the Atelidae family like *Cartelles* or *Caipora*, from the Pleistocene of Brazil (> 20 kg; Fig. 3; Supplementary Table S7 available on Dryad). Despite the expansion in range of platyrrhine body mass through time, most clades maintained intermediate sizes around 1–3 kg (Fig. 3). Most known fossil species until the Miocene, including all the taxa found in Patagonia, displayed intermediate body mass (e.g., pitheciids *Homunculus* and *Carlocebus* and cebins *Dolichocebus*, *Killikaike*; Supplementary Table S5 available on Dryad). Body size evolution in platyrrhines was largely heterogeneous, as indicated by several estimated rate shifts (Supplementary Table S10 available on Dryad), probably as a response to differential evolutionary selection forces acting on species across the vast range of Neotropical habitats.

Apart from the Late Miocene cebid *Acrecebus fraileyi* (12 kg), most large NWMs belong to the family Atelidae. The large body size of atelids is associated with particular locomotor adaptations, some of them displaying suspensory behavior, and all four living genera have prehensile tails (Fleagle 2013). Living atelids are widely distributed from Central America through northern Argentina, and some taxa can cope with open environments and seasonal climates, such as *Alouatta*. Despite the wide range of ecologies and adaptations in modern atelids and their large geographic range, the fossil record of the family does not include any specimen from the Miocene sites in Patagonia suggesting that they did not expand as far south as cebids and pitheciids (but see Tejedor 2002).

The smallest NWMs, the callitrichines (marmosets and tamarins), are characterized by a body mass smaller than 1 kg, a distribution spanning tropical forests from the Amazonia to Central America, and very scarce fossil record. The small body size of callitrichine has been usually considered as the result of evolutionary dwarfism (Ford 1980, 1986). This evolutionary trend is assumed to explain their atypical morphology, such as the reduction or loss of the third molars, loss of the hypocone, presence of claws instead of nails. However, in our analyses, we did not find evidence of a negative trend in body mass evolution within the subfamily (Supplementary Fig. S6 available on Dryad). Furthermore, the estimated size of the common ancestor of living callitrichines is small (around 0.6 kg; Fig. 3, Supplementary Table S11 available on Dryad), suggesting that callitrichines may have maintained a small body size throughout their evolutionary history. These results challenge the hypothesized dwarfism in callitrichines and suggest that their peculiar morphological features might be the result of ecological adaptations, which did not necessarily involve a reduction in body mass.

The process of body size evolution was heterogeneous across platyrrhine clades and did not follow a simple neutral evolution. We found evidence for several rate-shifts but only weak or negligible trends. Because, we incorporated topological uncertainties in our analyses, which is quite substantial for extinct lineages, it is difficult to pinpoint the exact placement of rate changes. However, we do observe that the rate of body mass evolution is highest at the family level and lower within most subfamilies (Supplementary Fig. S8 available on Dryad). This suggests a rapid change in body mass range as the three families diverged, between the Late Oligocene and the Early Miocene, followed by a slower pace in evolution within subfamilies (Supplementary Fig. S6 available on Dryad).

### Diversity Dynamics and Geographical Occupancy

Our analyses show that the Late Oligocene and Early Miocene also mark an important phase in the biogeography of NWMs, with a peak in fossil diversity and the widest latitudinal range in South America. The geographic expansion of platyrrhines started in the Late Oligocene and culminated between 20 and 15 Ma, when they reached their southernmost distribution. This geographic expansion is temporally coincident with a significant increase in NWMs population size as inferred in a recent analysis of genomic data (Schrago et al. 2014) and with a phase of positive net diversification rate (Fig. 5). Thus, both fossil and molecular data identify this phase as a crucial event in platyrrhine diversification and evolution.

During the Early Oligocene, the Patagonian mammal fauna had experienced a marked turnover (the “Patagonian Hinge”; Goin et al. 2012) associated with global climate cooling, during which Polydolopiformes, a diverse group of marsupials widely distributed in Patagonia and across South America, declined and eventually went extinct during the Early to Late Oligocene (Chornogubsky 2010, Goin et al. 2012). Among the Polydolopiformes, several groups showed evident primate-like adaptations in diet and probably paleoecology, and their extinction might have played a role facilitating the colonization of primates across Patagonia prior to the Early Miocene.

Climate change likely had a direct effect in determining NWMs’ changes in latitudinal range. Modern platyrrhines are mostly adapted to tropical environments and their occurrence in Patagonia around 20–16 Ma was likely allowed by the warmer climate characterizing the Miocene Climatic Optimum (MCO) (Zachos et al. 2008). Additionally, this period precedes a phase of major Andean uplift and the lack of a high elevation mountain range likely meant more humid paleoenvironments in the region, with moisture coming from the Pacific Ocean (Sepulchre et al. 2009). The MCO was followed by the diastrophic Quechua phase (Pascual and Ortíz Jaureguizar 1990), in which the regional climate and environment in the south of South America were affected both by global cooling and by the onset of Andean uplift. The latter progressively interrupted the influx of moisture from the Pacific and produced a rain shadow effect, thus turning Patagonia into a more open environment, with extensive grassland (Ortiz-Jaureguizar and Cladera 2006, Stromberg 2011) and causing a faunal turnover (Rosenberger et al. 2009, Palazzesi et al. 2014, Rohrmann et al. 2016). The environmental change that took place in Patagonia through the Middle and Late Miocene coincides with a range contraction of the southern limit of platyrrhine distribution (Fig. 4). The absence of latitudinal barriers (e.g., mountain ranges, deserts) in the continent has likely contributed to making these range expansions and contractions happen (Jaramillo and Cardenas 2013). Our results indicate that immediately after the MCO platyrrhines underwent a drop in their diversification rates and a spike in relative extinction (Fig. 5), suggesting that several Patagonian lineages have gone extinct after the MCO (Kay 2015, Herrera 2017). This extinction event in Patagonia is not limited to primates but extends across several mammalian lineages. In this major faunal turnover, temperate browsers and grazers diversified in the region, developing hypsodont dentition and larger size and replacing tropical or subtropical mammals such as sloths, anteaters, several arboreal marsupials, and arboreal caviomorphs (Rosenberger and Tejedor in press). The hypothesis that the diversification and the latitudinal range of platyrrhines have been strongly affected by climatic and environmental fluctuations is supported by a second event of range contraction at the onset of the Quaternary cooling and climatic fluctuations. This event also coincides with a sharp increase in relative extinction rates, although a decline in diversification rates towards the present is also consistent with a process of protracted speciation (Etienne and Rosindell 2012).

We infer a range expansion of platyrrhines towards the north of South America and into Central America and the Caribbean during the Miocene and Pliocene, although the scarcity of primate fossil records limits our ability to assess the detailed dynamics of this range expansion (Kay 2015). Nevertheless, our analyses, which included the recently described oldest record of NWM in North America (Supplementary Tables S4 and S5 available on Dryad) (Bloch et al. 2016), support the hypothesis of a Miocene colonization of the North American tropics coincident with a complex and progressive closure of the Panama Isthmus (Fig. 4) (Bacon et al. 2015a, Bacon et al. 2015b, Bloch et al. 2016).

### Methodological Advances

Recent studies have shown that the inclusion of fossils in phylogenetic analyses of trait evolution can improve dramatically our ability to infer ancestral states and the underlying evolutionary processes (Slater et al. 2013, Puttick 2016). Herein, we implemented a novel Bayesian statistical frameworks to integrate fossil and phylogenetic data in comparative analyses. We showed by simulations that the estimation of ancestral states in the presence of evolutionary trends is far from realistic unless at least some fossil information is provided (Fig. 2; Supplementary Figs. S1 and S2 available on Dryad).

Previous analyses of trait evolution combining data from extinct and extant taxa were based on node calibrations (Slater et al. 2012, Rolland et al. 2018), on the random *a posteriori *addition of extinct tips to a phylogenetic tree of extant taxa (Schnitzler et al. 2017), or on phylogenetic hypotheses built from morphological data (Slater 2015, Halliday and Goswami 2016, Slater et al. 2017). Node calibrations are based on the assumption that a fossil can be confidently placed at a node in the phylogeny of the living descendants, that is, it is not a stem or a side branch. The topological placement of fossils in a phylogeny (whether as nodal constraints or extinct tips) is difficult to determine in many clades especially when morphological traits that can be scored from fossils are scarce and bear little phylogenetic value, as in NWMs (Ronquist et al. 2012, Kay 2015). In our study, we used the available taxonomic information to constrain the position of fossil lineages and, at the same time, relied on the FBD process to sample multiple phylogenetic hypotheses based on an explicit process of speciation, extinction and preservation. Thus, by running trait-evolution analyses on FBD trees we incorporated topological and temporal uncertainties in our estimates (see Harrington and Reeder 2017 for an example on discrete traits).

Some limitations in inferring the evolutionary history of traits based on the fossil record remain. The small number and fragmentary nature of available fossil occurrences for most organism groups make it difficult to appreciate the amount of intraspecific variability (e.g., differences in body size across populations or linked to sexual dimorphism) and the impact of measurement error, both of which may affect the reliability of the estimates (Silvestro et al. 2015, Kostikova et al. 2016). In the case of biogeographic inferences, while more realistic and spatially explicit models (e.g., Lemmon and Lemmon 2008, Quintero et al. 2015, Villaaña and Rivadeneira 2018) would be desirable to infer the biogeographic history of a clade, the lack of extensive occurrence records means that analyses must rely on unavoidable simplifications and assumptions. For instance, most platyrrhine fossil taxa are known from single localities and we used these coordinates as representative of their latitudinal range, since an explicit correction of sampling biases in the fossil record requires a more extensive data set of fossil occurrences (Silvestro et al. 2016). Despite the limitations associated with the use of fossils in comparative analyses, paleontological data provide crucial evidence of traits that no longer exist in the living descendants (Slater et al. 2012). Furthermore, simulations using our Bayesian framework to infer trait evolution show that even very few fossils can drastically improve the estimation of ancestral states, indicating that our method can be applied to a large number of empirical data sets (Fig. 2). The importance of fossil information in the inference of evolutionary processes is evident in our analyses of body size and mean latitudinal range in platyrrhines, where the inclusion of fossil data significantly changes the estimated processes and ancestral states (Supplementary Figs. S8 and S9 and Tables S8 and S9 available on Dryad).

Our implementation of the EFBD process showed that the integration of living and extinct lineages allows us to infer negative diversification and complex temporal dynamics of speciation and extinction rates (Fig. 5). This is in line with recent studies demonstrating the benefits of incorporating extinct species in phylogenetic analyses of diversification rates (Didier et al. 2017, Gavryushkina et al. 2017). The importance of including fossil lineages in the estimation of speciation and extinction rates is apparent when comparing the results obtained from the platyrrhine phylogenetic tree pruned of the extinct lineages (Supplementary Fig. S11 available on Dryad). When fossils are excluded, the estimated extinction rates are low and essentially constant, the net diversification rates are always positive, and speciation rates appear to be highest in the Pliocene. A recent study of extant-taxa phylogenies and fossil record in primates showed that two data types often yielded contradictory results (Herrera 2017). Our EFBD estimates of negative diversification (i.e., diversity decline) following the MCO and in the Pleistocene (Fig. 5) match well with the trends of range contractions inferred from the latitudinal range analysis (Fig. 4) and coincide with phases of strong environmental change. These estimates are also much more comparable with those obtained using a non-phylogenetic method from fossil data only (Herrera 2017), which suggests they represent more realistic estimates.

## Conclusions

Our integrated analysis of molecular and fossil data of NWMs revealed that the stem platyrrhine lineage originated earlier than previously thought, and almost certainly in tropical regions. NWMs diversified shortly after their arrival from Africa into South America and quickly expanded their latitudinal range. This expansion coincided with a phase of positive net diversification and was probably favored by a global warming in the middle Miocene, which increased the extent of environmentally suitable regions for the diversification of tropical species such as most monkeys. Global cooling events after the middle Miocene and in the Quaternary, are associated with the disappearance of NWMs from the southernmost part of South America, resulting in a contraction of the range around the modern tropics and with increased relative extinction. Some primate populations living in seasonal environments are still distributed in northern Argentina, in open forests that may be analogous to the early Miocene Patagonian environments.

Platyrrhine evolution started with small forms, as shown by their Eocene North African relatives, but later diversified in a wide range of large and middle-sized taxa. In contrast, callitrichines kept small sizes throughout their evolutionary history, challenging the widely-accepted hypothesis of phyletic dwarfism in this clade. The evolution of body mass in platyrrhines overall appears to have followed a neutral process without strong trends, despite rate heterogeneity across branches. This pattern is potentially decoupled from the variable dynamics of diversity and latitudinal range, supporting the hypothesis that the diversification and distribution of primates in the New World are mostly driven by climate change, rather than by morphological adaptive evolution.

## Supplementary material

Data available from the Dryad Digital Repository: http://dx.doi.org/10.5061/dryad.sv43650.

## Appendix

### Platyrrhine Taxonomy

The taxonomic classification of Platyrrhini has been a matter of long standing debate among scholars. Recent investigations combining morphological traits and molecular data mostly converged on a classification that encompasses three families: Atelidae, Cebidae, and Pitheciidae, which we chose to follow in this study (Schneider 2000, Tejedor and Novo 2017). The basis of the current platyrrhine classification started with the morphological classification by Rosenberger (1980) and it was later supported by molecular analyses presented by Schneider et al. (1993). These studies led to a broad (albeit not universal) consensus about the taxonomy of platyrrhines, with the exception of the position of *Aotus* (discussed below; see also Schneider and Rosenberger 1996).

There remain, however, alternative classifications that split the platyrrhines into five families (e.g., Groves 2001, Rylands and Mittermeier 2009). Rylands and Mittermeier (2009) define five families, including Callitrichidae and the monotypic family Aotidae. However, callitrichines arguably need a more detailed and conclusive analysis of their relationships with other cebids, pitheciids, and atelids, before being confidently considered as a separate family. The definition of Aotidae as a separate family is problematic because the genus *Aotus* is consistently nested within Pitheciidae in phylogenetic analyses based on morphological data and within Cebidae in analyses of molecular data, including the present study (see Rosenberger and Tejedor 2013 and references therein for more details). While a full discussion of the debate around the taxonomy of platyrrhines goes beyond the scope of this study, we provide a summary of the different hypotheses in Supplementary Table S1 available on Dryad (see also Rosenberger 2011).

### Platyrrhine Fossil Placement

There are two main hypotheses about the placement of Patagonian platyrrhines in the platyrrhine phylogeny. One is the “Stem Hypothesis” (SH) and considers all Patagonian platyrrhines (except for *Proteropithecia*) as a separate lineage, namely the stem family Homunculinae (Kay 1990, 2015), for which, however, a clear morphological characterization (diagnosis) is currently lacking. The second hypothesis is the “Long Lineage Hypothesis” (LLH) and considers the Patagonian platyrrhines as part of the crown Platyrrhini, with several primitive representatives of modern families (Rosenberger 2010, Rosenberger and Tejedor 2013, Tejedor and Novo 2017). The latter hypothesis is supported by several phylogenetic analyses, which have consistently shown that the Patagonian primates are crown platyrrhines (Wilkinson et al. 2011, Perez et al. 2013, Bond et al. 2015). We, therefore, chose to follow the LLH when designing the topological constraints for fossil taxa (Supplementary Table S6 available on Dryad).

One of the fossil taxa included in our analyses, *Killikaike* (Tejedor et al. 2006), was synonymized with *Homunculus *by Perry et al. (2014). However, a thorough comparison between the skull of *Killikaike* and the *Homunculus* specimen MACN-A 5968 from the Ameghino collection (i.e., the only skull part of the hypodigm and attributed to *Homunculus* with certainty) showed substantial morphological differences between the two specimens (Tejedor and Novo 2017). We thus chose to retain *Killikaike* as a separate taxon.
